# Supplementary material for: Live imaging reveals the progenitors and cell dynamics of limb regeneration
Source: eLife. 2016 Oct 25;5:e19766. doi: 10.7554/eLife.19766 (PMC5079749; doi:10.7554/eLife.19766)
Supplement: Supplementary file 1. — DOI: http://dx.doi.org/10.7554/eLife.19766.020 [file elife-19766-supp1.docx]

Alwes, Enjolras and Averof (2016)

**Supplementary file 1**

Sequences for constructs *PhHS>lyn-TdTomato-2A-H2B-EGFP*, *DC5>DsRed* and *PhHS>EGFP-PhGemN*.

**A. *PhHS>lyn-TdTomato-2A-H2B-EGFP***

(from Eleni Kabrani, Diploma thesis, University of Crete, 2012)

LOCUS PhHS-lyn-TdTomato-2A-H2B-EGFP (AscI fragment) 5929 bp DNA linear 14/9/16

DEFINITION

ACCESSION

VERSION

SOURCE

ORGANISM

FEATURES Location/Qualifiers

misc_feature 42..2529

/label=PhHS regulatory sequence

CDS 2571..4121

/label=lyn-TdTomato coding sequence

CDS 4122..4184

/label=T2A ribosome skipping sequence

CDS 4203..5432

/label=H2B-EGFP coding sequence

misc_feature 5684..5915

/label=SV40 early polyA

ORIGIN

1 ggcgcgccTA GGCCGGCCGA ATTGGCCGCG GGAATTCGAT TTTACTGTAA CCGCAGGGGC

61 AAAAGAAGGC ATTCGAACGT GAGGCTGAGC AAACCAAACC CTTTACTTGA CTACCATATA

121 AAGTCTGTGA CAAGGTAGAA AATAAACTTT AAAAATAGCT GTAAGGTCTA CGGGATTTTT

181 TTTTTTCAAA TTTTTATCGG TGTTTTCCCC GTTATATATA GCCATTTTTT TTATCTTAAC

241 AGTGATTCCA TCAAAATTTA GACCATTTTG TTTCGAAATT CAATACTCTT TGAAAAAAAA

301 TGATAAGTTC TATAATCAGA AATTTTACTG GACATATATG GCTTTCATCA TTTCAACGAA

361 AAATCACTGA AATATTTCTT ATGTTGTACC TGAAACTCTT GGGTCTCACA ATATGCAATG

421 CGGTTGCGTT CAATAAGATC TTATTTTTAC AAAGATTGAT CATTCTCCTT ATAAGAATAT

481 CATGAAAAAT GTTAAAATAA TTAACAAGAT CCAAACAGAC GTCATATTCT TTACAAGTTG

541 GAAGTTATCT CAATATTATT CGAAGTATAA GTTTATAAAT TTTATTTTGC TTCATTCATA

601 CACTATCGTA ACTTTTTATG TAGCCATACA GTATATTTAT TGAGTACATG TGTGCACGAT

661 GCCCGTGAAC GAACTAGCCT ACTTGAGTGT TTGGTAATTT TCATTGACGA AATTATTCGC

721 CAGCGTCATA GCCCAGACTC AAAGAGCATT CCTGTATAGA ATTCAATGTT ATTTTATTAT

781 TCCTAGAATC ACTGATACAT ATTTACATTT ACAGGATGCT ATAGACCGTC CTGCAACATT

841 CCCGAACATT CGTTTTCTTC TACGGCATTC TCGAAAATTC GAGAATGCTC GGCGTGAAGG

901 GAGACTGAGA TATAAGAAGC TGCTTCGCCG CCAGCCTCTC AGTTTATCCT GAGCTTCTAA

961 TCGCGATACA TACTACCATT TTGCTCACAC GAAAGTAGAG AAAGGACTGG AATTTATTCT

1021 GTACCCTGTG AATAATTTGC TGAAGTGTAG TTATTGTCAA GAAAACCAAT TTTATACTGT

1081 TTGATACCTG TTCGAAAGAC GCGAGTTCTG CGAAGTGATT TTTTAAGTCC TCTACATCAC

1141 TTCAGCGCTA CTGTGTTTGT TATTTGAAGG TAAGCTTGAA TTGAATTGCA TTATGTTGCT

1201 AAATTTTGTG TAAACTGAAG CTTAAGGAAC TGATTAGGGC TCTGTAGCTA CTAGTACAGC

1261 TGTTCTTCTT TCTGAAGCAA TTATTTTCCT AAATGGTGGA AAACAGTTAC ATATTTATCA

1321 ACATGGCAAA AGCTGAAAAG AAAAAAAAAA AATTTCTAAA TAATTTATTT ATTCCTTCAA

1381 CTTCAGGCAT TTTATTCTTC AAGAATAACC TTCAGGAAAA TTACATTCAG ATGTTTTCAA

1441 CTGAGACGTA ACAAACTGTC TTTCATGCCA AGAGCCAAAA TTGTCTCATT ATATCCAACA

1501 GTTCAGAATA AAAAGTTCAG TGAAAGAAGT ACGATCGTTT AAGTTTGAAT ATTGTTTACT

1561 AATAAGTAAT TGATTAATAT ACTGACCAAA GTAACTAAAA TAGAATAGAT TGGTAAACAA

1621 TAACTGTGAG TGTCAACTCT GAGACTGTAT AAGCTCTGTC TGCTGTTTTA TATATGTAAA

1681 TGAGTTTGAA ATTACGACAC TGAATTATGA ACAATGTGCG TTAGCGAGTG TACTGAGCTA

1741 TGAAGCTGCT ATTGCTTCAT TCCAAAATTC TACGATCGCT GGCCGTGAGT GTTGACTTAC

1801 AGTAGGAAGT GTGTGGTTTA CCCGAGATTC AGTTATAATG TGTGTCTATA CATACCTTAA

1861 GTTAGACACC TATTTCTATC TTACCCCAAC CTATATATCC TATTCTAACC TAACCCAACA

1921 CTCTAAGTTA ACCAGACCTA ATAGGCTGGG TTACTACGTT TCGAATATTT AGTACCTTTG

1981 TAAAAATCGT CAGAAATTGC GCGGGCGCCT ACAATGGAGG TCTCTGTTAT TTCATTGATG

2041 TTTTAGTCCT TCAAGCCGTG CAAGGATTGG ACTTGTAGCC GAGTCTACAT TTGCCAAACT

2101 CTTCCCATTA TACATTTGTA AAATTCTGCT GGTGAACTGC TTAATATTCT GAAAAGTTTA

2161 TTTATGCAAC TGCACAAATC ACGTTGGTTC TCTTTATTTC CAATGAAGGA AAAAATCGAT

2221 TTCCTGCAAT TTGGGTGAAT TACTTGAATA ATACTAAGAA ATTCGTGACT GGAAGCTGTG

2281 TAATGGCTTT CGGCAGTAAA TATTTGTATT TTCAGTGCAG TTACACTTCC TAAAATACTG

2341 AACATAAAAA CGGCAGAATG CAAATTACTG CAAATCTTTT GAAGAAAATA ATTATTCATG

2401 TCATGGAAGC GTAAGTGGAA CATCAATTTA TTAATTATCT TGTGAATATT TATTGTTCAT

2461 AATTTACAAT CGATTTTCTT TTAGTGACGT TGCTTATTAT GTTTTTTGTT CACAGAACAA

2521 TCCACAACCG GCCGCGGGAA TTCAAGGCCT CTCGAGCCTC TAGAGCCACC ATGGGCTGCA

2581 TCAAGAGCAA GCGCAAGGAC AACCTGAACG ACGACGAGGC CGCCATGGGC TGCATCAAGA

2641 GCAAGCGCAA GGACAACCTG AACGACGACG AGGCACCGGA AGATCCCGCC ACCATGGTGA

2701 GCAAGGGCGA GGAGGTCATC AAAGAGTTCA TGCGCTTCAA GGTGCGCATG GAGGGCTCCA

2761 TGAACGGCCA CGAGTTCGAG ATCGAGGGCG AGGGCGAGGG CCGCCCCTAC GAGGGCACCC

2821 AGACCGCCAA GCTGAAGGTG ACCAAGGGCG GCCCCCTGCC CTTCGCCTGG GACATCCTGT

2881 CCCCCCAGTT CATGTACGGC TCCAAGGCGT ACGTGAAGCA CCCCGCCGAC ATCCCCGATT

2941 ACAAGAAGCT GTCCTTCCCC GAGGGCTTCA AGTGGGAGCG CGTGATGAAC TTCGAGGACG

3001 GCGGTCTGGT GACCGTGACC CAGGACTCCT CCCTGCAGGA CGGCACGCTG ATCTACAAGG

3061 TGAAGATGCG CGGCACCAAC TTCCCCCCCG ACGGCCCCGT AATGCAGAAG AAGACCATGG

3121 GCTGGGAGGC CTCCACCGAG CGCCTGTACC CCCGCGACGG CGTGCTGAAG GGCGAGATCC

3181 ACCAGGCCCT GAAGCTGAAG GACGGCGGCC ACTACCTGGT GGAGTTCAAG ACCATCTACA

3241 TGGCCAAGAA GCCCGTGCAA CTGCCCGGCT ACTACTACGT GGACACCAAG CTGGACATCA

3301 CCTCCCACAA CGAGGACTAC ACCATCGTGG AACAGTACGA GCGCTCCGAG GGCCGCCACC

3361 ACCTGTTCCT GGGGCATGGC ACCGGCAGCA CCGGCAGCGG CAGCTCCGGC ACCGCCTCCT

3421 CCGAGGACAA CAACATGGCC GTCATCAAAG AGTTCATGCG CTTCAAGGTG CGCATGGAGG

3481 GCTCCATGAA CGGCCACGAG TTCGAGATCG AGGGCGAGGG CGAGGGCCGC CCCTACGAGG

3541 GCACCCAGAC CGCCAAGCTG AAGGTGACCA AGGGCGGCCC CCTGCCCTTC GCCTGGGACA

3601 TCCTGTCCCC CCAGTTCATG TACGGCTCCA AGGCGTACGT GAAGCACCCC GCCGACATCC

3661 CCGATTACAA GAAGCTGTCC TTCCCCGAGG GCTTCAAGTG GGAGCGCGTG ATGAACTTCG

3721 AGGACGGCGG TCTGGTGACC GTGACCCAGG ACTCCTCCCT GCAGGACGGC ACGCTGATCT

3781 ACAAGGTGAA GATGCGCGGC ACCAACTTCC CCCCCGACGG CCCCGTAATG CAGAAGAAGA

3841 CCATGGGCTG GGAGGCCTCC ACCGAGCGCC TGTACCCCCG CGACGGCGTG CTGAAGGGCG

3901 AGATCCACCA GGCCCTGAAG CTGAAGGACG GCGGCCACTA CCTGGTGGAG TTCAAGACCA

3961 TCTACATGGC CAAGAAGCCC GTGCAACTGC CCGGCTACTA CTACGTGGAC ACCAAGCTGG

4021 ACATCACCTC CCACAACGAG GACTACACCA TCGTGGAACA GTACGAGCGC TCCGAGGGCC

4081 GCCACCACCT GTTCCTGTAC GGCATGGACG AGCTGTACAA GGGCAGTGGA GAGGGCAGAG

4141 GAAGTCTGCT AACATGCGGT GACGTCGAGG AGAATCCTGG CCCAGGAGAT CTGAATATCA

4201 CCATGCCAGA GCCAGCGAAG TCTGCTCCCG CCCCGAAAAA GGGCTCCAAG AAGGCGGTGA

4261 CTAAGGCGCA GAAGAAAGGC GGCAAGAAGC GCAAGCGCAG CCGCAAGGAG AGCTATTCCA

4321 TCTATGTGTA CAAGGTTCTG AAGCAGGTCC ACCCTGACAC CGGCATTTCG TCCAAGGCCA

4381 TGGGCATCAT GAATTCGTTT GTGAACGACA TTTTCGAGCG CATCGCAGGT GAGGCTTCCC

4441 GCCTGGCGCA TTACAACAAG CGCTCGACCA TCACCTCCAG GGAGATCCAG ACGGCCGTGC

4501 GCCTGCTGCT GCCTGGGGAG TTGGCCAAGC ACGCCGTGTC CGAGGGTACT AAGGCCGTCA

4561 CCAAGTACAC CAGCGCTAAG GGATCTGGGG GTGGAGGCTC CGGCGGGGGT GGATCTGGAG

4621 GTGGGGGCAG CGGCGGAGGT GGGCTAGCGC TACCGCGGGC CACCATGAGC AAGGGCGAGG

4681 AGCTGTTCAC CGGGGTGGTG CCCATCCTGG TCGAGCTGGA CGGCGACGTA AACGGCCACA

4741 AGTTCAGCGT GTCCGGCGAG GGCGAGGGCG ATGCCACCTA CGGCAAGCTG ACCCTGAAGT

4801 TCATCTGCAC CACCGGCAAG CTGCCCGTGC CCTGGCCCAC CCTCGTGACC ACCCTGACCT

4861 ACGGCGTGCA GTGCTTCAGC CGCTACCCCG ACCACATGAA GCAGCACGAC TTCTTCAAGT

4921 CCGCCATGCC CGAAGGCTAC GTCCAGGAGC GCACCATCTT CTTCAAGGAC GACGGCAACT

4981 ACAAGACCCG CGCCGAGGTG AAGTTCGAGG GCGACACCCT GGTGAACCGC ATCGAGCTGA

5041 AGGGCATCGA CTTCAAGGAG GACGGCAACA TCCTGGGGCA CAAGCTGGAG TACAACTACA

5101 ACAGCCACAA CGTCTATATC ATGGCCGACA AGCAGAAGAA CGGCATCAAG GCCAACTTCA

5161 AGATCCGCCA CAACATCGAG GACGGCAGCG TGCAGCTCGC CGACCACTAC CAGCAGAACA

5221 CCCCCATCGG CGACGGCCCC GTGCTGCTGC CCGACAACCA CTACCTGAGC ACCCAGTCCG

5281 CCCTGAGCAA AGACCCCAAC GAGAAGCGCG ATCACATGGT CCTGCTGGAG TTCGTGACCG

5341 CCGCCGGGAT CACTCACGGC ATGGACGAGC TGTACAAGTC CGGACTCAGA TCATCAAGCT

5401 TATCGGATCC GGGCGGCCGT GATAATAGGT GATAGTAAGT AATAGTGAGA TATCTCTAGA

5461 ATTCCAGAAC AACAACAATT GCATTCATTT TATGTTTCAG GTTCAGGGGG AGGTGTGGGA

5521 GGTTTTTTAA TTCGCGGCCT GGCCAAGAAG CCCGTGCAGC TGCCCGGCTA CTACTACGTG

5581 GACTCCAAGC TGGACATCAC CTCCCACAAC GAGGACTACA CCATCGTGGA GCAGTACGAG

5641 CGCGCCGAGG GCCGCCACCA CCTGTTCCTG TAGCGGCCGC GACTCTAGAT CATAATCAGC

5701 CATACCACAT TTGTAGAGGT TTTACTTGCT TTAAAAAACC TCCCACACCT CCCCCTGAAC

5761 CTGAAACATA AAATGAATGC AATTGTTGTT GTTAACTTGT TTATTGCAGC TTATAATGGT

5821 TACAAATAAA GCAATAGCAT CACAAATTTC ACAAATAAAG CATTTTTTTC ACTGCATTCT

5881 AGTTGTGGTT TGTCCAAACT CATCAATGTA TCTTAAAGCT Tggcgcgcc

//

**B. *DC5>DsRed***

(from Nikolaos Konstantinides, Masters thesis, University of Crete, 2008)

LOCUS DC5-DsRed (AscI fragment) 3135 bp DNA linear 14/9/16

DEFINITION

ACCESSION

VERSION

SOURCE

ORGANISM

FEATURES Location/Qualifiers

misc_feature 73..450

/label=DC5 regulatory sequence

misc_feature 558..2201

/label=Part of PhHS regulatory sequence (lacking HSF binding sites)

CDS 2202..2879

/label=DsRed.T1 coding sequence

misc_feature 2890..3121

/label=SV40 early polyA

ORIGIN

1 ggcgcgccTA GGCCGGCCGA ATTGGCCGCG GTGGCGGCCG CTCTAGAACT AGTGGATCCC

61 CCGGGCTGCA GGAATTCAGC TTCCATGGCT GTAGCAGCTT GCATGCCTGC AGGTCGACAG

121 ATCTAAATAT TCATTGTTGT TGCTCACCTA CCATGGATCT AAATATTCAT TGTTGTTGCT

181 CACCTACCAT GGATCTAAAT ATTCATTGTT GTTGCTCACC TACCATGGAT CTAAATATTC

241 ATTGTTGTTG CTCACCTACC ATGGATCTAA ATATTCATTG TTGTTGCTCA CCTACCATGG

301 ATCTAAATAT TCATTGTTGT TGCTCACCTA CCATGGATCT AAATATTCAT TGTTGTTGCT

361 CACCTACCAT GGATCTAAAT ATTCATTGTT GTTGCTCACC TACCATGGAT CCTCGACTCT

421 AGAGGATCCC CGGGTACCGA GCTCGAATTC GATATCAAGC TTATCGATAC CGTCGACCTC

481 GAGGGGGGGC CCGGTACCTC TAGAACTATA GCTAGCATGC GCAAATTTAA AGCGCTGATA

541 TCGATCGCGC GCAGATCTGC TCGGCGTGAA GGGAGACTGA GATATAAGAA GCTGCTTCGC

601 CGCCAGCCTC TCAGTTTATC CTGAGCTTCT AATCGCGATA CATACTACCA TTTTGCTCAC

661 ACGAAAGTAG AGAAAGGACT GGAATTTATT CTGTACCCTG TGAATAATTT GCTGAAGTGT

721 AGTTATTGTC AAGAAAACCA ATTTTATACT GTTTGATACC TGTTCGAAAG ACGCGAGTTC

781 TGCGAAGTGA TTTTTTAAGT CCTCTACATC ACTTCAGCGC TACTGTGTTT GTTATTTGAA

841 GGTAAGCTTG AATTGAATTG CATTATGTTG CTAAATTTTG TGTAAACTGA AGCTTAAGGA

901 ACTGATTAGG GCTCTGTAGC TACTAGTACA GCTGTTCTTC TTTCTGAAGC AATTATTTTC

961 CTAAATGGTG GAAAACAGTT ACATATTTAT CAACATGGCA AAAGCTGAAA AGAAAAAAAA

1021 AAAATTTCTA AATAATTTAT TTATTCCTTC AACTTCAGGC ATTTTATTCT TCAAGAATAA

1081 CCTTCAGGAA AATTACATTC AGATGTTTTC AACTGAGACG TAACAAACTG TCTTTCATGC

1141 CAAGAGCCAA AATTGTCTCA TTATATCCAA CAGTTCAGAA TAAAAAGTTC AGTGAAAGAA

1201 GTACGATCGT TTAAGTTTGA ATATTGTTTA CTAATAAGTA ATTGATTAAT ATACTGACCA

1261 AAGTAACTAA AATAGAATAG ATTGGTAAAC AATAACTGTG AGTGTCAACT CTGAGACTGT

1321 ATAAGCTCTG TCTGCTGTTT TATATATGTA AATGAGTTTG AAATTACGAC ACTGAATTAT

1381 GAACAATGTG CGTTAGCGAG TGTACTGAGC TATGAAGCTG CTATTGCTTC ATTCCAAAAT

1441 TCTACGATCG CTGGCCGTGA GTGTTGACTT ACAGTAGGAA GTGTGTGGTT TACCCGAGAT

1501 TCAGTTATAA TGTGTGTCTA TACATACCTT AAGTTAGACA CCTATTTCTA TCTTACCCCA

1561 ACCTATATAT CCTATTCTAA CCTAACCCAA CACTCTAAGT TAACCAGACC TAATAGGCTG

1621 GGTTACTACG TTTCGAATAT TTAGTACCTT TGTAAAAATC GTCAGAAATT GCGCGGGCGC

1681 CTACAATGGA GGTCTCTGTT ATTTCATTGA TGTTTTAGTC CTTCAAGCCG TGCAAGGATT

1741 GGACTTGTAG CCGAGTCTAC ATTTGCCAAA CTCTTCCCAT TATACATTTG TAAAATTCTG

1801 CTGGTGAACT GCTTAATATT CTGAAAAGTT TATTTATGCA ACTGCACAAA TCACGTTGGT

1861 TCTCTTTATT TCCAATGAAG GAAAAAATCG ATTTCCTGCA ATTTGGGTGA ATTACTTGAA

1921 TAATACTAAG AAATTCGTGA CTGGAAGCTG TGTAATGGCT TTCGGCAGTA AATATTTGTA

1981 TTTTCAGTGC AGTTACACTT CCTAAAATAC TGAACATAAA AACGGCAGAA TGCAAATTAC

2041 TGCAAATCTT TTGAAGAAAA TAATTATTCA TGTCATGGAA GCGTAAGTGG AACATCAATT

2101 TATTAATTAT CTTGTGAATA TTTATTGTTC ATAATTTACA ATCGATTTTC TTTTAGTGAC

2161 GTTGCTTATT ATGTTTTTTG TTCACAGAAC AATCCACAAC CATGGCCTCC TCCGAGGACG

2221 TCATCAAGGA GTTCATGCGC TTCAAGGTGC GCATGGAGGG CTCCGTGAAC GGCCACGAGT

2281 TCGAGATCGA GGGCGAGGGC GAGGGCCGCC CCTACGAGGG CACCCAGACC GCCAAGCTGA

2341 AGGTGACCAA GGGCGGCCCC CTGCCCTTCG CCTGGGACAT CCTGTCCCCC CAGTTCCAGT

2401 ACGGCTCCAA GGTGTACGTG AAGCACCCCG CCGACATCCC CGACTACAAG AAGCTGTCCT

2461 TCCCCGAGGG CTTCAAGTGG GAGCGCGTGA TGAACTTCGA GGACGGCGGC GTGGTGACCG

2521 TGACCCAGGA CTCCTCCCTG CAGGACGGCT CCTTCATCTA CAAGGTGAAG TTCATCGGCG

2581 TGAACTTCCC CTCCGACGGC CCCGTAATGC AGAAGAAGAC TATGGGCTGG GAGGCCTCCA

2641 CCGAGCGCCT GTACCCCCGC GACGGCGTGC TGAAGGGCGA GATCCACAAG GCCCTGAAGC

2701 TGAAGGACGG CGGCCACTAC CTGGTGGAGT TCAAGTCCAT CTACATGGCC AAGAAGCCCG

2761 TGCAGCTGCC CGGCTACTAC TACGTGGACT CCAAGCTGGA CATCACCTCC CACAACGAGG

2821 ACTACACCAT CGTGGAGCAG TACGAGCGCG CCGAGGGCCG CCACCACCTG TTCCTGTAGC

2881 GGCCGCGACT CTAGATCATA ATCAGCCATA CCACATTTGT AGAGGTTTTA CTTGCTTTAA

2941 AAAACCTCCC ACACCTCCCC CTGAACCTGA AACATAAAAT GAATGCAATT GTTGTTGTTA

3001 ACTTGTTTAT TGCAGCTTAT AATGGTTACA AATAAAGCAA TAGCATCACA AATTTCACAA

3061 ATAAAGCATT TTTTTCACTG CATTCTAGTT GTGGTTTGTC CAAACTCATC AATGTATCTT

3121 AAAGCTTggc gcgcc

//

**C. *PhHS>EGFP-PhGemN***

LOCUS PhHS-EGFP-PhGemN (AscI_fragment) 3961 bp DNA linear 14/9/16

DEFINITION

ACCESSION

VERSION

SOURCE

ORGANISM

FEATURES Location/Qualifiers

misc_feature 42..2529

/label=PhHS regulatory sequence

CDS 2530..3705

/label=EGFP-PhGemN coding sequence

misc_feature 3716..3947

/label=SV40 early polyA

ORIGIN

1 ggcgcgccTA GGCCGGCCGA ATTGGCCGCG GGAATTCGAT TTTACTGTAA CCGCAGGGGC

61 AAAAGAAGGC ATTCGAACGT GAGGCTGAGC AAACCAAACC CTTTACTTGA CTACCATATA

121 AAGTCTGTGA CAAGGTAGAA AATAAACTTT AAAAATAGCT GTAAGGTCTA CGGGATTTTT

181 TTTTTTCAAA TTTTTATCGG TGTTTTCCCC GTTATATATA GCCATTTTTT TTATCTTAAC

241 AGTGATTCCA TCAAAATTTA GACCATTTTG TTTCGAAATT CAATACTCTT TGAAAAAAAA

301 TGATAAGTTC TATAATCAGA AATTTTACTG GACATATATG GCTTTCATCA TTTCAACGAA

361 AAATCACTGA AATATTTCTT ATGTTGTACC TGAAACTCTT GGGTCTCACA ATATGCAATG

421 CGGTTGCGTT CAATAAGATC TTATTTTTAC AAAGATTGAT CATTCTCCTT ATAAGAATAT

481 CATGAAAAAT GTTAAAATAA TTAACAAGAT CCAAACAGAC GTCATATTCT TTACAAGTTG

541 GAAGTTATCT CAATATTATT CGAAGTATAA GTTTATAAAT TTTATTTTGC TTCATTCATA

601 CACTATCGTA ACTTTTTATG TAGCCATACA GTATATTTAT TGAGTACATG TGTGCACGAT

661 GCCCGTGAAC GAACTAGCCT ACTTGAGTGT TTGGTAATTT TCATTGACGA AATTATTCGC

721 CAGCGTCATA GCCCAGACTC AAAGAGCATT CCTGTATAGA ATTCAATGTT ATTTTATTAT

781 TCCTAGAATC ACTGATACAT ATTTACATTT ACAGGATGCT ATAGACCGTC CTGCAACATT

841 CCCGAACATT CGTTTTCTTC TACGGCATTC TCGAAAATTC GAGAATGCTC GGCGTGAAGG

901 GAGACTGAGA TATAAGAAGC TGCTTCGCCG CCAGCCTCTC AGTTTATCCT GAGCTTCTAA

961 TCGCGATACA TACTACCATT TTGCTCACAC GAAAGTAGAG AAAGGACTGG AATTTATTCT

1021 GTACCCTGTG AATAATTTGC TGAAGTGTAG TTATTGTCAA GAAAACCAAT TTTATACTGT

1081 TTGATACCTG TTCGAAAGAC GCGAGTTCTG CGAAGTGATT TTTTAAGTCC TCTACATCAC

1141 TTCAGCGCTA CTGTGTTTGT TATTTGAAGG TAAGCTTGAA TTGAATTGCA TTATGTTGCT

1201 AAATTTTGTG TAAACTGAAG CTTAAGGAAC TGATTAGGGC TCTGTAGCTA CTAGTACAGC

1261 TGTTCTTCTT TCTGAAGCAA TTATTTTCCT AAATGGTGGA AAACAGTTAC ATATTTATCA

1321 ACATGGCAAA AGCTGAAAAG AAAAAAAAAA AATTTCTAAA TAATTTATTT ATTCCTTCAA

1381 CTTCAGGCAT TTTATTCTTC AAGAATAACC TTCAGGAAAA TTACATTCAG ATGTTTTCAA

1441 CTGAGACGTA ACAAACTGTC TTTCATGCCA AGAGCCAAAA TTGTCTCATT ATATCCAACA

1501 GTTCAGAATA AAAAGTTCAG TGAAAGAAGT ACGATCGTTT AAGTTTGAAT ATTGTTTACT

1561 AATAAGTAAT TGATTAATAT ACTGACCAAA GTAACTAAAA TAGAATAGAT TGGTAAACAA

1621 TAACTGTGAG TGTCAACTCT GAGACTGTAT AAGCTCTGTC TGCTGTTTTA TATATGTAAA

1681 TGAGTTTGAA ATTACGACAC TGAATTATGA ACAATGTGCG TTAGCGAGTG TACTGAGCTA

1741 TGAAGCTGCT ATTGCTTCAT TCCAAAATTC TACGATCGCT GGCCGTGAGT GTTGACTTAC

1801 AGTAGGAAGT GTGTGGTTTA CCCGAGATTC AGTTATAATG TGTGTCTATA CATACCTTAA

1861 GTTAGACACC TATTTCTATC TTACCCCAAC CTATATATCC TATTCTAACC TAACCCAACA

1921 CTCTAAGTTA ACCAGACCTA ATAGGCTGGG TTACTACGTT TCGAATATTT AGTACCTTTG

1981 TAAAAATCGT CAGAAATTGC GCGGGCGCCT ACAATGGAGG TCTCTGTTAT TTCATTGATG

2041 TTTTAGTCCT TCAAGCCGTG CAAGGATTGG ACTTGTAGCC GAGTCTACAT TTGCCAAACT

2101 CTTCCCATTA TACATTTGTA AAATTCTGCT GGTGAACTGC TTAATATTCT GAAAAGTTTA

2161 TTTATGCAAC TGCACAAATC ACGTTGGTTC TCTTTATTTC CAATGAAGGA AAAAATCGAT

2221 TTCCTGCAAT TTGGGTGAAT TACTTGAATA ATACTAAGAA ATTCGTGACT GGAAGCTGTG

2281 TAATGGCTTT CGGCAGTAAA TATTTGTATT TTCAGTGCAG TTACACTTCC TAAAATACTG

2341 AACATAAAAA CGGCAGAATG CAAATTACTG CAAATCTTTT GAAGAAAATA ATTATTCATG

2401 TCATGGAAGC GTAAGTGGAA CATCAATTTA TTAATTATCT TGTGAATATT TATTGTTCAT

2461 AATTTACAAT CGATTTTCTT TTAGTGACGT TGCTTATTAT GTTTTTTGTT CACAGAACAA

2521 TCCACAACCA TGGTGAGCAA GGGCGAGGAG CTGTTCACCG GGGTGGTGCC CATCCTGGTC

2581 GAGCTGGACG GCGACGTAAA CGGCCACAAG TTCAGCGTGT CCGGCGAGGG CGAGGGCGAT

2641 GCCACCTACG GCAAGCTGAC CCTGAAGTTC ATCTGCACCA CCGGCAAGCT GCCCGTGCCC

2701 TGGCCCACCC TCGTGACCAC CCTGACCTAC GGCGTGCAGT GCTTCAGCCG CTACCCCGAC

2761 CACATGAAGC AGCACGACTT CTTCAAGTCC GCCATGCCCG AAGGCTACGT CCAGGAGCGC

2821 ACCATCTTCT TCAAGGACGA CGGCAACTAC AAGACCCGCG CCGAGGTGAA GTTCGAGGGC

2881 GACACCCTGG TGAACCGCAT CGAGCTGAAG GGCATCGACT TCAAGGAGGA CGGCAACATC

2941 CTGGGGCACA AGCTGGAGTA CAACTACAAC AGCCACAACG TCTATATCAT GGCCGACAAG

3001 CAGAAGAACG GCATCAAGGT GAACTTCAAG ATCCGCCACA ACATCGAGGA CGGCAGCGTG

3061 CAGCTCGCCG ACCACTACCA GCAGAACACC CCCATCGGCG ACGGCCCCGT GCTGCTGCCC

3121 GACAACCACT ACCTGAGCAC CCAGTCCGCC CTGAGCAAAG ACCCCAACGA GAAGCGCGAT

3181 CACATGGTCC TGCTGGAGTT CGTGACCGCC GCCGGGATCA CTCTCGGCAT GGACGAGCTG

3241 TACAAGGGAT ATCCTGCAGG CTGGCGACCG CTCGAGATGG TTGAACGTAA AAAGTTAGGG

3301 GTGTTGCAGC CCACTCAGCA GTGCAGGCTT ACTGTGCACC AGTCTAAGAT TATGGTGGAT

3361 TCACCTAAGG CTCTGAAACG CAAAGCCTTC GGTGATGAAA ACTTCTCGCC TGTCCCCACA

3421 CTGAGCCGGA AGTCTATCTT TGAGGACCCA CCAACACCCG CATCGAAGCG CTCCAAACTT

3481 TCTGATAAAG TGGATTCTGC TATTCAGACT GACAATCTGT GTTGTGGTAA TGGCGGTACA

3541 GAAATCTCAC GAGGGTCTTC ATCTTCCAAA TCTTCGTCAG AAGCATCTAC TAAAGCATGC

3601 TCTCAGAAAT CCCAATCCCA AAGCGTTCTG GACATGTTAA CAAGTGCAGA GCCAAATGAG

3661 CAGTACTGGC AGATGATGGC TGCAGAACGG AAAGCTGCGC TCTAGCGGCC GCGACTCTAG

3721 ATCATAATCA GCCATACCAC ATTTGTAGAG GTTTTACTTG CTTTAAAAAA CCTCCCACAC

3781 CTCCCCCTGA ACCTGAAACA TAAAATGAAT GCAATTGTTG TTGTTAACTT GTTTATTGCA

3841 GCTTATAATG GTTACAAATA AAGCAATAGC ATCACAAATT TCACAAATAA AGCATTTTTT

3901 TCACTGCATT CTAGTTGTGG TTTGTCCAAA CTCATCAATG TATCTTAAAG CTTggcgcgc

3961 c

//
